# Peptidylarginine Deiminase 2 in Murine Antiviral and Autoimmune Antibody Responses

**DOI:** 10.1155/2022/5258221

**Published:** 2022-01-17

**Authors:** Aisha M. Mergaert, Michael F. Denny, Brock Kingstad-Bakke, Mandar Bawadekar, S. Janna Bashar, Thomas F. Warner, Marulasiddappa Suresh, Miriam A. Shelef

**Affiliations:** ^1^Department of Medicine, University of Wisconsin-Madison, Madison, WI, USA; ^2^Department of Pathology and Laboratory Medicine, University of Wisconsin-Madison, Madison, WI, USA; ^3^Department of Pathobiological Sciences, University of Wisconsin-Madison, Madison, WI, USA; ^4^William S. Middleton Memorial Veterans Hospital, Madison, WI, USA

## Abstract

The peptidylarginine deiminases (PADs) and the citrullinated proteins that they generate have key roles in innate immunity and rheumatoid arthritis, an inflammatory arthritis with antibodies that target citrullinated proteins. However, the importance of PADs, particularly PAD2, in the adaptive immune response, both normal and pathogenic, is newly emerging. In this study, we evaluated a requirement for PAD2 in the antibody response in collagen-induced arthritis (CIA), a T and B cell-driven murine model of rheumatoid arthritis, and in the protective antibody response to murine influenza infection. Using PAD2^−/−^ and PAD2^+/+^ mice on the DBA/1J background, we found that PAD2 is required for maximal anti-collagen antibody levels, but not collagen-specific plasma cell numbers, T cell activation or polarization, or arthritis severity in CIA. Also, we found that PAD2 is required not just for normal levels of persistent hemagglutination inhibiting antibodies but also for full protection from lethal influenza rechallenge. Together, these data provide evidence for a novel modest requirement for PAD2 in a normal antiviral antibody response and in an abnormal autoantibody response in inflammatory arthritis.

## 1. Introduction

The peptidylarginine deiminases (PADs) are essential components of normal and pathologic inflammation. They catalyze citrullination, the posttranslational modification of arginine to the nonstandard amino acid citrulline. PAD2 and PAD4 are expressed in hematopoietic cells [1–3], and many studies have evaluated their roles in innate immunity and inflammation. Most prominently, extensive evidence suggests that PAD4, more than PAD2, contributes to the formation of citrullinated neutrophil extracellular traps [4–6], which have been implicated in a wide range of normal and pathologic immune responses [7]. More recent work demonstrates a requirement for the PADs in adaptive immune responses, and PAD2 appears to play a prominent role. It is required for production of IL-17A, but not IFN*γ*, by T cells from imiquimod-treated, but not untreated, mice [8] with a similar requirement for PAD2 in IL-17A production by *in vitro* differentiated Th17 cells [3]. PAD2 is also required to suppress IL-4 production by Th2-differentiated T cells, but has no effect on IFN*γ* production by Th1-differentiated cells [3].

PAD2 and PAD4 also appear to be important in rheumatoid arthritis, an autoimmune arthritis with autoantibodies that target citrullinated antigens [9]. PAD2 and PAD4 are present in the rheumatoid joint [10], have genetic variants associated with rheumatoid arthritis risk [11–14], and are required for full arthritis severity in TNF-induced arthritis [5, 15], a murine model of innate inflammation in arthritis. Unlike in human rheumatoid arthritis, citrullinated antigens are not specifically targeted by antibodies in murine TNF-induced arthritis [15], suggesting that these two PADs provide distinct contributions to immune cell function in inflammatory arthritis. Although the exact mechanisms of these contributions are unclear, reductions in serum IgG levels and plasma cell numbers in PAD2-deficient mice with TNF-induced arthritis suggest involvement of lymphocytes [5].

Despite accumulating evidence for the importance of PAD2 for T and B cell function, a requirement for PAD2 has not been evaluated in an IL-17-driven [16] model of rheumatoid arthritis with pathogenic autoantibodies [17], such as collagen-induced arthritis (CIA), whereas PAD4 was shown to be required for full arthritis severity in this model [18]. Moreover, PAD2 has not been studied in a normal adaptive immune response to a virus, such as the antibody-dependent response to influenza [19]. PAD4 was dispensable in an influenza response *in vivo* [20], but PAD2^−/−^ mice have more prominent antibody defects than PAD4^−/−^ mice [5, 15]. Such studies are important to reveal potential benefits and consequences of PAD inhibition, since PAD inhibitors have been proposed as therapeutic agents for the treatment of diseases in which citrullination is thought to be pathogenic, including rheumatoid arthritis, lupus, cardiovascular disease, thrombosis, multiple sclerosis, and cancer [21, 22].

In this manuscript, we evaluate the role of PAD2 in an abnormal autoantibody response in a murine model of rheumatoid arthritis and in a protective antibody response using a murine model of influenza infection. We find that PAD2 is required for maximal persistent levels of anti-collagen antibodies in CIA, but not for T cell activation or polarization, or arthritis severity. PAD2 is also required for maximal levels of hemagglutination-inhibiting antibodies at key time points and full protection from influenza.

## 2. Materials and Methods

### 2.1. Mice

PAD2^−/−^ mice [23] backcrossed 12 generations to the DBA/1J background [4] and age- and sex-matched DBA/1J PAD2^+/+^ controls were used for all experiments. Similar numbers of male and female mice were used. Mice of both genotypes were cohoused whenever possible, and when not, bedding was mixed for CIA experiments. Absence of PAD2 was confirmed in several PAD2^−/−^ mice by western blot of bone marrow lysates. Mice were maintained in specific-pathogen-free conditions, and experiments were approved by the University of Wisconsin Animal Care and Use Committee.

### 2.2. Collagen-Induced Arthritis Induction

Arthritis was induced in 7-8-week-old mice by intradermal tail injection of 50-100 *μ*l of an emulsion of equal parts 1 mg/ml chick type II collagen (Chondrex, Redmond, USA) and complete Freund's adjuvant (CFA, BD, Franklin Lakes, USA) followed by intraperitoneal injection of 100 *μ*l of an emulsion of equal parts 1 mg/ml chick type II collagen with incomplete Freund's adjuvant (IFA, BD) 21 and 42 days after the initial injection. After initial injection, mice were fed a high fat diet (2019 diet, Teklad, Madison, USA).

### 2.3. Influenza Infection

To determine optimum infection conditions, mice were infected intranasally with 100, 33, 10, or 3 plaque forming units (PFU) of A/PR/8/34 H1N1 [24] or 50 or 10 PFU of A/PR/8/34 H1N1–OT-I (PR8-OVA) [24, 25] in PBS. Influenza virus was prepared in the laboratory of Professor Yoshihiro Kawaoka (University of Wisconsin-Madison). For PAD2^−/−^ experiments, 8-9-week-old mice were infected with 10 PFU of PR8-OVA in 50 *μ*l of PBS. After 3 months, mice were rechallenged with 3,000 PFU of PR8 in 50 *μ*l of PBS. Mice were weighed daily and monitored for health for 14 days after each infection. Mice with severe disease defined by 20%-30% weight loss were euthanized.

### 2.4. Enzyme-Linked Immunosorbent Assay (ELISA)

Flat bottom high bind 96-well plates (Corning, Tewksbury, USA) were incubated with 40 ng/ml PR8 hemagglutinin (HA, BEI Resources, Manassas, USA) in PBS or with bovine collagen (Chondrex) at 10 *μ*g/ml in 50 mM carbonate-bicarbonate buffer overnight at 4°C. Wells were washed with wash buffer (0.1% Tween 20 in PBS) followed by blocking with 5% nonfat dried milk with 2% fetal bovine serum (FBS, Atlanta Biologicals, Flowery Branch, USA) in wash buffer (HA block) or with 1% BSA in wash buffer (collagen block) for 2 hours at room temperature (RT). For collagen ELISA, serum was applied to the plate at a 1 : 10,000 dilution in collagen block and incubated at 4°C overnight, followed by washing, then application of a 1 : 5000 dilution of goat anti-mouse IgG Fc conjugated to horseradish peroxidase (HRP, Southern Biotech, Birmingham, USA). For HA ELISA, serum was applied to the plate at 1 : 1000 (anti-IgG ELISA) or 1 : 200 (anti-IgM and IgA) dilutions in HA block and incubated at RT for 1 hour. Then, plates were washed, followed by incubation for 1 hour at RT with dilutions of 1 : 10,000 goat anti-mouse IgG Fc-HRP (Southern Biotech), 1 : 5000 goat anti-mouse IgM-HRP (Southern Biotech), or 1 : 1000 goat anti-mouse IgA Fc-HRP (LSBio, Seattle, USA) in PBS. All plates were then washed, developed with Slow-TMB ELISA solution (Thermo Fisher, Waltham, USA), stopped with 0.2 M H_2_SO_4_, and read using a Synergy 2 plate reader (BioTek, Winooski, USA) equipped with Gen5.0 software or a FilterMAX F3 plate reader equipped with SoftMax Pro software (Molecular Devices, San Jose, USA). The 540 nm signal was subtracted from the 450 nm signal, and values for blank wells were subtracted.

To standardize anti-collagen ELISAs, a standard curve was performed on each plate using serially diluted (from 1 : 1000 to 1 : 1024000) CIA reference serum (Hooke Labs, Lawrence, USA). The standard curve was used to convert experimental sample absorbance values into a relative value compared to the CIA reference.

For HA ELISA, absorbance values were converted to ng/ml of Ig using a standard curve. Each plate included a standard curve, which was generated by coating wells with streptavidin, followed by 5 *μ*g/ml biotin conjugated goat anti-mouse IgG (Mabtech, Nacka Strand, Sweden), rabbit anti-mouse IgM (Mabtech), or rat anti-mouse IgA (Biolegend, San Diego, USA). Serial dilutions of purified mouse IgG (Jackson ImmunoResearch, West Grove, USA) or IgM (Millipore, Burlington, USA) ranging from 0.0169 to 1000 ng/ml or IgA (Invitrogen, Carlsbad, USA) ranging from 0.0085 to 500 ng/ml were applied to appropriate wells followed by the same steps as above. After correcting for background, absorbance values for mouse sera were converted to ng/ml of Ig using a four-parameter nonlinear curve fit for the standard curve wells (http://elisaanalysis.com/).

### 2.5. Enzyme-Linked Immune Absorbent Spot (ELISpot)

Bone marrow cells were flushed from mouse femurs under aseptic conditions with sterile PBS, dispersed by several passages through a 21 gauge needle, and strained through a 100-micron nylon mesh filter, followed by immersion in 0.8% NH_4_Cl red blood cell lysis solution (Stemcell Technologies, Vancouver, Canada). Cells were resuspended in B cell media (RPMI 1260 with 10% FBS, 50 *μ*M beta-mercaptoethanol, L-glutamine, streptomycin, and penicillin). PVDF filter plates were prepared per manufacturer's instructions (Millipore). Wells were incubated overnight at 4°C with 10 *μ*g/ml bovine type II collagen, washed with PBS, and blocked with RPMI 1640 (Thermo Fisher Scientific) with 2% FBS, penicillin, streptomycin, and 50 *μ*M beta-mercaptoethanol at 37°C for 2 hours. Bone marrow cells in B cell media were applied in triplicate to wells and incubated overnight in a humidified incubator at 37°C with 5% CO_2_. Plate membranes were then washed with PBS, then with wash buffer, then incubated with 0.5 *μ*g/ml biotinylated goat anti-mouse IgG (Mabtech) in PBS with 1% BSA and 0.2% Tween 20 (ELISpot wash) for 2 hours at RT or overnight at 4°C, washed with wash buffer, incubated with avidin-HRP at 1 : 10,000 in wash buffer for 1 hour at RT, washed twice and developed with fresh 2 mg/ml aminoethylcarbazole in acetate solution for up to 20 minutes, neutralized with tap water, and left to dry. Spots were counted by eye in a blinded manner.

### 2.6. Limiting-Dilution Assay (LDA)

Bone marrow cell suspensions of 2.5 ml each at densities of 2.0 × 10^6^, 1.33 × 10^6^, 0.5 × 10^6^, and 0.2 × 10^6^ cells/ml in B cell media were prepared from the femurs of each mouse. A round bottom 96-well tissue culture plate was filled by aseptically transferring 0.1 ml of each suspension density into a total of 24 wells. The plates containing the bone marrow cells in culture were incubated for 20 hours at 37°C in a humidified 10% CO_2_ tissue culture incubator, with each plate corresponding to one mouse sample. To detect wells containing collagen-specific antibody secreting cells, a corresponding 96-well collagen ELISA plate was prepared. RIA/EIA binding plates (Corning) were coated overnight at 4°C with 10 *μ*g/ml chick collagen type II in 50 mM carbonate-bicarbonate buffer (pH > 8.0). ELISA wells were washed and blocked for 2 hours at RT with collagen block. The blocking buffer was removed, and 85 *μ*l of the conditioned B cell media from the overnight incubation of bone marrow cells was directly transferred into the corresponding well of the collagen ELISA plate. The collagen ELISA plates were incubated and developed as described above for the anti-collagen ELISA, except the collagen standard curve for relative titer determination was omitted. In nonimmunized DBA1/J mice, a background subtracted OD450 of 0.03 is approximately 9 standard deviations above mean background absorbance for negative wells. The frequency of anti-collagen type II antibody secreting cells at each cell density was calculated based upon the Poisson distribution of limiting dilution analysis. The number of antibody secreting cells was calculated for each cell density using the equation *m* = −ln(*F*_0_), where *m* is the number of antibody secreting cells and *F*_0_ is the fraction of wells that are negative for anti-collagen reactivity. A linear regression was applied to the graph of anti-collagen IgG-producing bone marrow cell number versus plated bone marrow cell density, and the frequency of antibody secreting cells calculated by solving for the cell density at the point where ln(*F*_0_) = −0.368.

### 2.7. Flow Cytometry

Splenocytes were resuspended in a buffer of 1% BSA, 2% FBS, 0.03% sodium azide, and 2 mM EDTA in PBS for staining with CD8b-Alexa Fluor 488 (H35-17.2, Invitrogen), CD4-PE-CF594 (RM4-5, BD), CD62L-PE-Cy7 (MEL-14, Tonbo Biosciences, San Diego, USA), CD44-Brilliant Violet 421 (IM7, Biolegend), CD3e-APC (145-2C11, Biolegend), and/or ghost dye (Red 780, Tonbo Biosciences) and were fixed in 2% paraformaldehyde in PBS after staining. Helper T cell characterization was performed using a mouse Th1/Th2/Th17 Phenotyping Kit (BD Biosciences, San Jose, USA) per the manufacturer's instructions. Samples were acquired using a LSRII machine, and data were analyzed using FlowJo software (BD Biosciences).

### 2.8. Arthritis Scoring

Paws were scored weekly by A.M.M. in a blinded manner for range of motion (normal 0, reduced in digits 1, reduced in wrist/ankle or more 2), usage (normal 0, abnormal weight bearing or loss of grip strength 1, nonuse 2), swelling (none 0, any digit 1, paw 2, wrist/ankle 3, entire limb 4), and erythema (none 0, slight 1, extreme 2). Each limb was scored according to each parameter and scores for all limbs averaged for each mouse to create a final score. Paw thickness was measured with a digital caliper (SPI, Melville, USA) weekly with measurements of all paws averaged for a final measurement.

### 2.9. Histology

Front paws were fixed and decalcified in Decalcifier I (Leica, Buffalo Grove, USA), embedded in paraffin, sectioned, and stained with hematoxylin and eosin. Carpal, carpometacarpal, metacarpophalangeal, and interphalangeal joints were scored on a scale of 0 to 8 by T.F.W. in a blinded manner for each of the following characteristics: pannus with synovitis, cartilage destruction, and ankylosis. Scores were averaged for each mouse.

### 2.10. Hemagglutinin Inhibition (HI) Assay

Sera were treated with receptor destroying enzyme II (Denka Seiken, Tokyo, Japan) per the manufacturer's instructions, then mixed with 4 HA units of PR8 virus in a round bottom 96-well plate (Corning). After a 30-minute incubation at 37°C, 0.5% chicken red blood cells (Lampire Biological Laboratories, Pipersville, USA) in PBS were added and mixed, then incubated for a minimum of 30 minutes at RT. The lowest titer at which hemagglutination of red blood cells occurred was recorded.

### 2.11. Statistics

A paired *t* test was used for all analyses with *p* < 0.05 considered significant.

## 3. Results

**Figure 1:**
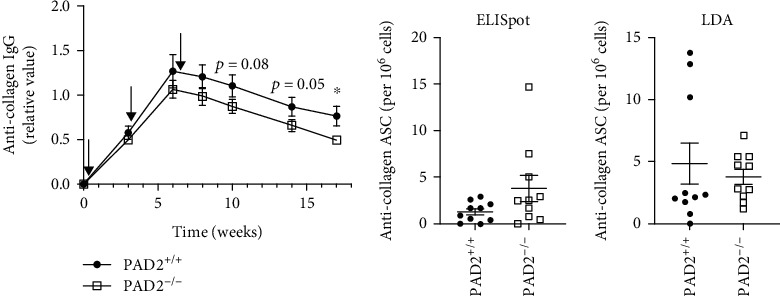
In collagen-induced arthritis (CIA), PAD2 is required for normal anti-collagen IgG levels, but not numbers of anti-collagen antibody secreting cells. CIA was induced in PAD2^+/+^ and PAD2^−/−^ mice on the DBA/1J background with the first injection at week zero. (a) Sera were collected at indicated time points post-CIA induction and anti-collagen IgG levels quantified by ELISA and expressed as a value relative to a CIA serum standard (mean ± SEM, *n* = 23 mice, 6 experiments). Gray arrows indicate CIA-inducing injections. Bone marrow-derived anti-collagen IgG antibody secreting cells (ASCs) were quantified by (b) ELISpot and (c) anti-collagen ELISA limiting dilution assay (LDA) 17 weeks post-CIA induction (symbols indicate ASC numbers in individual mice, horizontal bar indicates mean ± SEM, *n* = 10 mice, 3 experiments). For all panels, PAD2^+/+^ versus PAD2^−/−^ data were compared by paired *t* test and ^∗^*p* < 0.05.

To evaluate the role of PAD2 in a model of rheumatoid arthritis driven by T cells and anti-collagen autoantibodies, we induced CIA in PAD2^+/+^ and PAD2^−/−^ mice on the DBA/1J background. Since IgG levels were reduced in the absence of PAD2 in TNF-induced arthritis [5], we evaluated a requirement for PAD2 in anti-collagen IgG levels by ELISA. As shown in Figure 1(a), anti-collagen IgG increased in all mice, but the elevated levels did not persist as strongly in PAD2^−/−^ as compared to PAD2^+/+^ mice, with a significantly lower level at 17 weeks post-CIA induction. Next, we evaluated the number of anti-collagen IgG-producing cells in the bone marrow at 17 weeks post-CIA induction as measured by ELISpot and limiting dilution ELISA assay [26]. No difference in the number of anti-collagen IgG-producing cells could be detected between PAD2^−/−^ and PAD2^+/+^ mice (Figures 1(b) and 1(c)).

**Figure 2:**
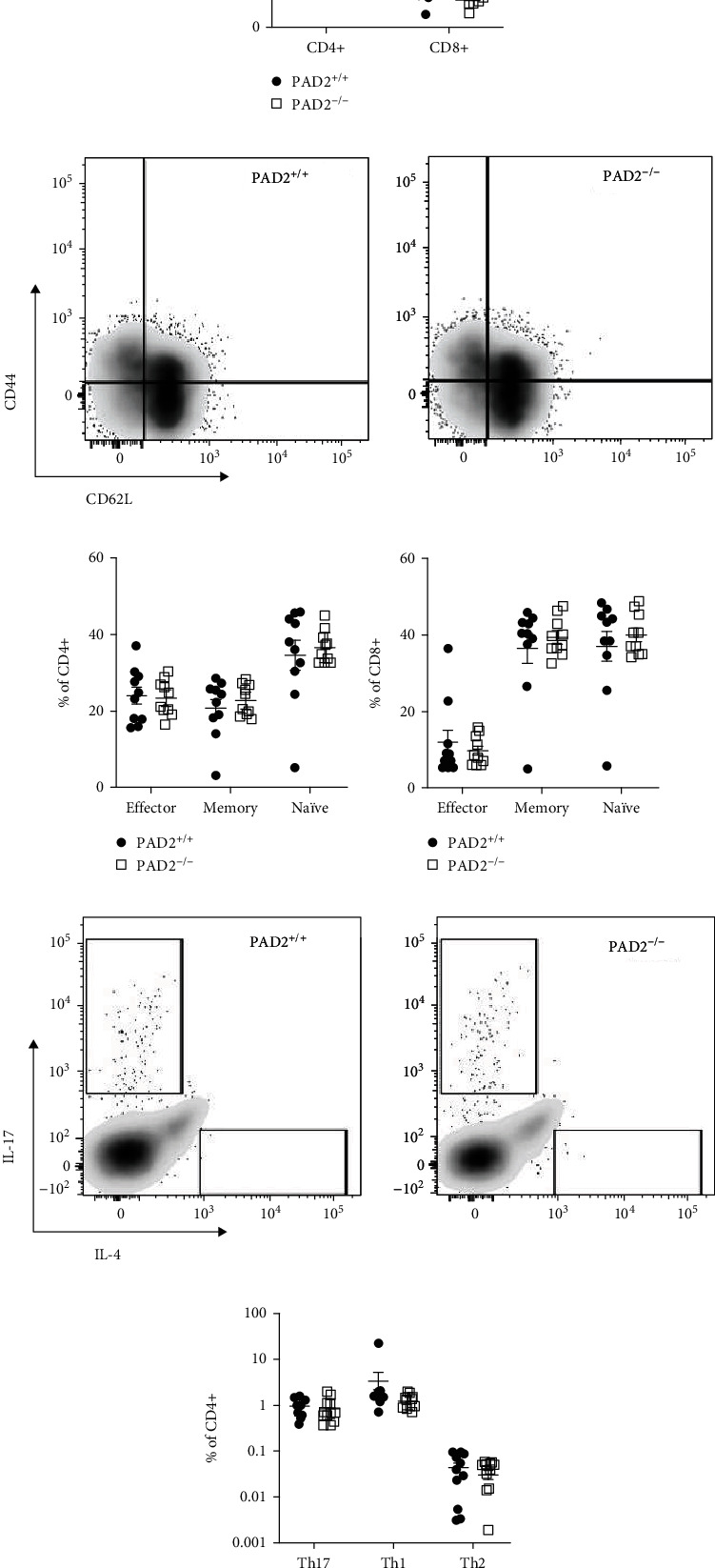
T cells in collagen-induced arthritis (CIA) are unaffected by loss of PAD2. Seventeen weeks after the first injection to induce CIA, splenocytes were harvested and stained for flow cytometry. Debris, clustered cells, and dead cells were excluded using forward scatter, side scatter, and a viability dye. (a) Percent of CD3+ splenocytes that are CD4+ and CD8+ (*n* = 10 mice, 3 experiments). Activation status of these CD3+ cells was evaluated with (b) representative plots as well as mean percent ± SEM of (c) CD4+ and (d) CD8+ populations that are effector (CD62L-CD44+), memory (CD62L+CD44+), and naïve (CD62L+CD44-) shown. The percent of CD4+ cells that produce IL-17A, IL-4, and IFN*γ* was also quantified with (e) representative plots and (f) mean percent ± SEM graphed (*n* = 12 mice, 3 experiments). For all panels, PAD2^+/+^ versus PAD2^−/−^ data were compared by paired *t* test, and no comparisons were significant.

Given the role of PAD2 in IL-17 production, we next evaluated a requirement for PAD2 in the T cell compartment in CIA. PAD2^−/−^ and PAD2^+/+^ mice had similar numbers of CD3+ T cells in the spleen 17 weeks after the first CIA injection (16.8 × 10^6^ ± 11.7 × 10^6^ and 16.9 × 10^6^ ± 8 × 10^6^, respectively). Moreover, there was no difference in the percent of CD3+ cells that were CD4+ or CD8+ (Figure 2(a)) or the frequencies of naïve, effector, or memory CD4+ or CD8+ T cells (Figures 2(b)–2(d)). Finally, PAD2^−/−^ mice did not have altered frequencies of CD4+ cells that produced IL-17, IL-4, or IFN*γ* (Figures 2(e) and 2(f)).

**Figure 3:**
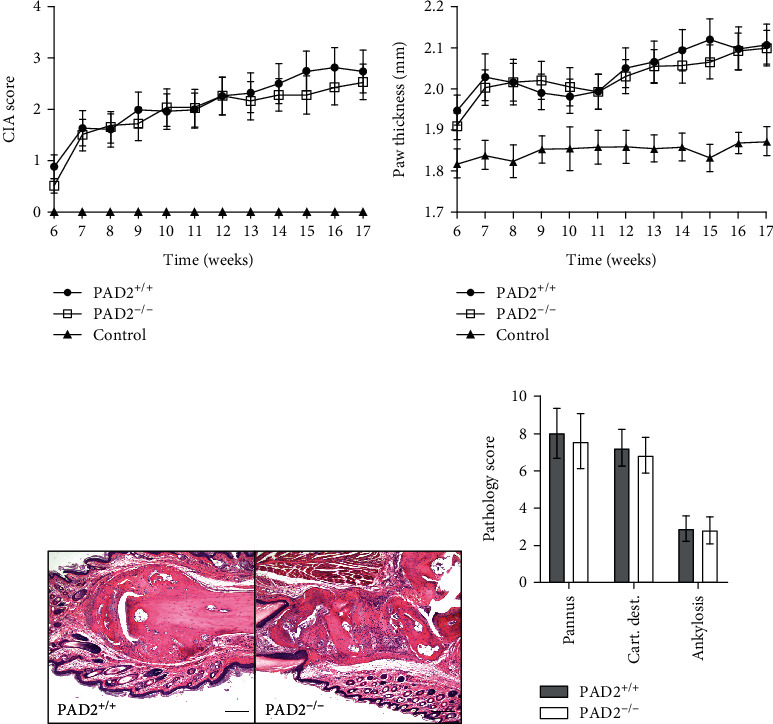
Arthritis severity in collagen-induced arthritis (CIA) is unaltered in the absence of PAD2. Six weeks after the first injection to induce CIA, (a) clinical scores and (b) paw thickness were assessed weekly (*n* = 23 mice, 6 experiments). Controls (*n* = 9), which received no injections and were cohoused with experimental mice, were also evaluated. At 17 weeks after the first injection, the front paws were fixed, embedded, sectioned, and stained with hematoxylin and eosin. (c) Representative images at 100x. Bar indicates 200 microns. (d) The extent of pannus development, cartilage destruction, and ankylosis were scored in a blinded manner (*n* = 23 mice, 6 experiments). All graphs depict mean ± SEM. PAD2^+/+^ versus PAD2^−/−^ data were compared by paired *t* test, and no comparisons were significant.

Next, we evaluated arthritis severity in the absence of PAD2. Clinical scores and paw thickness were determined weekly for 12 weeks starting on the date of the final CIA injection. As shown in Figures 3(a) and 3(b), PAD2^−/−^ mice did not have reduced arthritis compared to PAD2^+/+^ mice by either measurement. Additionally, paws were analyzed for histological differences and showed that synovitis, cartilage destruction, and ankylosis at 17 weeks postinitial injection were unaltered in the absence of PAD2 (Figures 3(c)and 3(d)). Taken together, these data suggest a modest requirement for PAD2 for persistently high levels of anti-collagen IgG in CIA, but not for the number of anti-collagen IgG-secreting cells, T cell activation or polarization, or arthritis severity.

**Figure 4:**
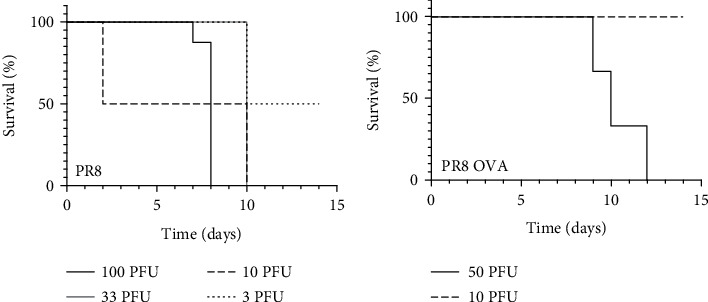
DBA/1J mice are highly susceptible to influenza infection. Mice were infected with different doses of PR8 or PR8-OVA followed by daily health monitoring. Kaplan–Meier curves depict survival of DBA/1J mice after (a) PR8 infection (100 PFU *n* = 8, 33.3 PFU *n* = 2, 10 PFU *n* = 2, 3 PFU *n* = 2) or (b) PR8-OVA infection (50 PFU *n* = 3, 10 PFU *n* = 2).

Given the lower anti-collagen IgG levels in PAD2-deficient mice late in CIA as well as the importance of protective antibodies for immunity against influenza [19], we wanted to assess the role of PAD2 in antibody-based immunity to PR8, a mouse-adapted H1N1 *influenza* virus. However, we could find no reports of the use of PR8 in DBA/1 J mice. Thus, we first evaluated viral dosing in the DBA/1 J strain. We infected a small number of DBA/1 J mice with a range of PFUs of PR8 to start to determine optimal dosing. However, as shown in Figure 4(a), even extremely low PR8 doses could be lethal to DBA/1 J mice. Thus, we switched to PR8-OVA, a modified PR8 virus with the SIINFEKL peptide of chicken ovalbumin (i.e., OVA) in its neuraminidase stalk, since the insertion of short peptides into influenza neuraminidase, including the OVA peptide, can reduce viral growth *in vivo* [25, 27]. Despite the reduced virulence, we still observed lethality in DBA/1J mice from 50 PFU, so we selected the very low dose of 10 PFU of PR8-OVA, which was not lethal in DBA/1J mice in our small study, for further experiments (Figure 4(b)).

**Figure 5:**
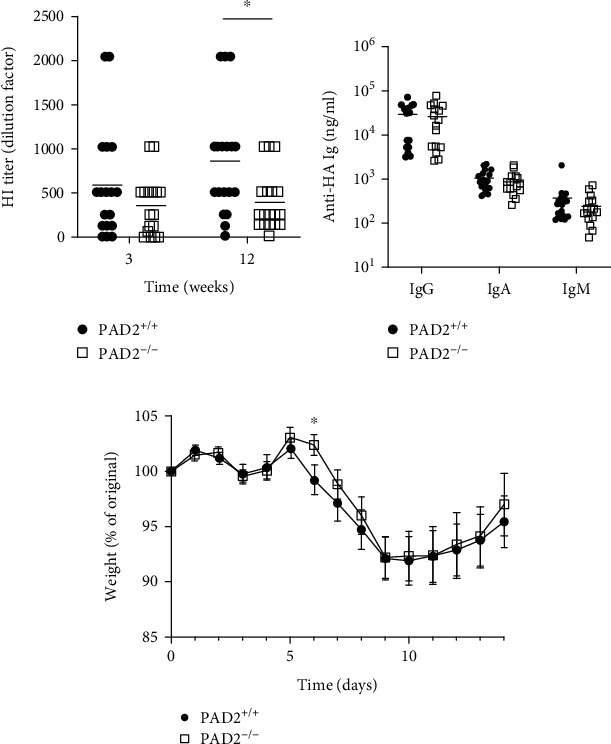
PAD2 is required for hemagglutination inhibiting antibody titers in DBA/1J mice after influenza infection. PAD2^+/+^ and PAD2^−/−^ mice on the DBA/1J background were infected with PR8-OVA. (a) Sera were collected at the indicated time points and used in a hemagglutination inhibition (HI) assay (*n* = 18, 5 experiments). (b) Sera at 12 weeks postinfection were used to detect anti-hemagglutinin (HA) IgG, IgA, and IgM by ELISA (*n* = 17‐18). (c) Body weights were obtained daily for 14 days postinfection and reported as percent of weight on the day of infection (*n* = 18, 5 experiments). For all panels, PAD2^+/+^ versus PAD2^−/−^ data were compared by paired *t* test, graphs depict mean ± SEM, and ^∗^*p* < 0.05.

We next evaluated the requirement for PAD2 in a primary response to influenza infection. PAD2^+/+^ and PAD2^−/−^ mice were infected with 10 PFU of PR8-OVA, and serum was collected prior to infection as well as 3 and 12 weeks after infection to quantify hemagglutination inhibiting antibodies by HI assay and anti-HA antibodies by ELISA. No hemagglutination inhibiting antibodies were detected in any mouse before exposure to PR8-OVA. As shown in Figure 5(a), PAD2^−/−^ mice had equivalent HI titers at 3 weeks postinfection, but reduced titers compared to PAD2^+/+^ mice at 12 weeks postinfection. There was no corresponding reduction of anti-HA IgG, IgA, or IgM levels in PAD2^−/−^ mice at 12 weeks postinfection (Figure 5(b)). We also evaluated weight loss, a measure of influenza disease severity, in the PAD2^+/+^ and PAD2^−/−^ mice after infection and saw essentially no difference in weight loss between PAD2^+/+^ and PAD2^−/−^ mice (Figure 5(c)).

**Figure 6:**
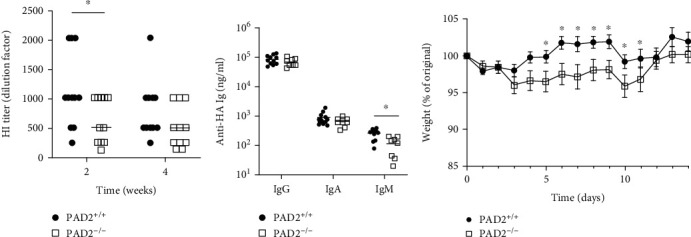
PAD2 is required for normal protection from influenza reinfection. PAD2^+/+^ and PAD2^−/−^ mice on the DBA/1J background were infected with PR8-OVA, followed by lethal rechallenge 12 weeks later with PR8. (a) Sera were collected at the indicated time points and used in a hemagglutination inhibition (HI) assay (*n* = 11, 3 experiments). (b) Sera from two weeks postchallenge were used to detect anti-hemagglutinin (HA) IgG, IgA, and IgM by ELISA (*n* = 9‐11). (c) Body weights were obtained daily for 14 days postchallenge infection and reported as percent of weight on the day of challenge infection (*n* = 11, 3 experiments). For all panels, PAD2^+/+^ versus PAD2^−/−^ data were compared by paired *t* test, graphs depict mean ± SEM, and ^∗^*p* < 0.05.

Given the reduced HI titers at 12 weeks postinfection with PR8-OVA and the importance of hemagglutination-inhibiting antibodies for protective immunity against influenza [19], we next evaluated the role of PAD2 in a rechallenge influenza infection using a viral dose of PR8 lethal to naïve DBA/1J mice. Twelve weeks after initial infection with PR8-OVA, we rechallenged PAD2^+/+^ and PAD2^−/−^ mice with 3,000 PFU of PR8 and performed the same assays as above. We found that PAD2^−/−^ mice had reduced HI titers compared to PAD2^+/+^ mice two weeks after challenge, with a corresponding reduction in anti-HA IgM levels (Figures 6(a) and 6(b)). Moreover, PAD2^−/−^ mice had twice as much weight loss and required three times longer to return to their starting weight than PAD2^+/+^ mice after the lethal rechallenge (Figure 6(c)). Taken together, these data suggest that PAD2 is required for normal antibody titers late in a primary antiviral response and early in a reinfection response as well as for full protection from influenza reinfection in DBA/1J mice.

## 4. Discussion

In this manuscript, we demonstrate a modest, yet novel, role for PAD2 in persistent anti-collagen antibody levels in a murine model of rheumatoid arthritis and in normal levels of hemagglutination inhibiting antibodies, as well as for full protection from influenza in DBA/1J mice.

In response to growing evidence of the importance of PAD2 for B and T lineage cells, this study is the first to evaluate the role of PAD2 in CIA. A pan-PAD inhibitor was shown to reduce CIA severity [28], but this may have been due to inhibition of multiple PADs or potentially just PAD4, since arthritis severity is reduced in PAD4-deficient mice with CIA [18]. In this manuscript, we show that PAD2 is not required for arthritis severity in CIA (Figure 3). In contrast, we previously identified a requirement for PAD2 in TNF-induced arthritis severity [5]. Thus, PAD2 may be important in the innate immune cells thought to be the predominant drivers of TNF-induced arthritis, with a less critical role in the adaptive immune processes in CIA. Although not required for neutrophil extracellular trap formation [4, 5], PAD2 is required for optimal inflammasome assembly and IL-1*β* release by macrophages [29]. Since TNF can drive IL-1*β* secretion via the NLRP3 inflammasome [30], the role of PAD2 in TNF-induced arthritis may be macrophage-dependent. We did observe a reduction of anti-collagen antibodies (Figure 1(a)), which are known to be able to drive arthritis [17], but the ~35% reduction of anti-collagen antibodies late in disease in PAD2^−/−^ mice may have been insufficient to reduce arthritis severity in a detectable manner in CIA, a model with notoriously high variability.

Additionally, PAD2 was seemingly dispensable in the T cell compartment at 17 weeks postinduction of CIA (Figure 2). The absence of a requirement for PAD2 in Th17 cells in CIA may have contributed to the lack of a detectable reduction in arthritis in PAD2-deficient mice. In contrast, previous studies demonstrated a requirement for PAD2 in Th17 cells [3, 8], a T cell subset important in CIA [16, 31, 32]. The reason for the discrepant findings is unknown. Perhaps PAD2 is only required in some contexts for Th17 polarization and IL-17 production, such as in the imiquimod-mediated model of lupus [8] or in *in vitro*-differentiated Th17 cells [3], but not in Th17 cells in CIA. In CIA, the multiple injections of collagen plus adjuvant might overcome mild defects in Th17 cells due to loss of PAD2. Timing may also be a factor. We examined T cell responses about four months after CIA induction, while other studies evaluated Th17 cells earlier in disease [31, 33, 34]. However, even if Th17 responses were altered in PAD2^−/−^ mice earlier in CIA, there was no effect on arthritis severity.

In contrast to the lack of a detectable role for PAD2 in Th17 cells in CIA, PAD2 was required for maximal levels of persistent anti-collagen antibodies (Figure 1(a)). This finding is consistent with reduced antibodies in PAD2-deficient mice with TNF-induced arthritis [5]. Curiously, numbers of anti-collagen IgG secreting cells were unaltered in PAD2^−/−^ mice, suggesting either low sensitivity of the assays that quantify these cells, or that the reduced anti-collagen IgG resulted from other mechanisms such as lower IgG production in each cell or an alteration of IgG half-life in PAD2^−/−^ mice.

We also found that PAD2 was required for maximal levels of hemagglutination inhibiting antibodies in response to influenza infection at key time points (Figures 5 and 6). The reduction in HI titers did not always correspond with a reduction in total levels of anti-HA Ig (Figure 5). While both tests measure antiviral antibodies, the HI assay quantifies functional antibodies, which typically correlate with protective immunity, while the anti-HA ELISA measures total antibody levels, irrespective of functional capacity. Thus, the differences seen between the two assays could reflect inherent caveats in assay sensitivities or a decrease in the relative amount of functional antibodies within the total anti-HA antibody repertoire [35, 36]. However, two weeks after the lethal challenge infection, anti-HA IgM levels were reduced in PAD2-deficient mice. Although IgG is typically considered the Ig isotype of long-lived plasma cells conferring protection from reinfection, influenza-specific neutralizing IgM antibodies can persist for 18 months and protect mice from influenza-mediated death [37, 38].

Consistent with reduced HI titers in PAD2^−/−^ mice, protection from influenza virus-induced weight loss upon viral reinfection was reduced in the absence of PAD2, a novel role for PAD2 or any PAD. PAD4-deficient mice did not lose more weight than wild type mice after primary infection with influenza A/WSN/33/H1N1 or have any detected reduced ability to respond to influenza, but rechallenge infection was not evaluated [20]. Moreover, antibody defects in PAD2^−/−^ mice are more profound than PAD4^−/−^ mice [5, 15], potentially explaining the role for PAD2 and not PAD4 in an antiviral response. Further studies to determine the mechanism by which PAD2 regulates protection from influenza are needed in a genetic background for which there are more immunological tools, such as C57BL/6 mice.

Of note, in order to demonstrate a role for PAD2 in influenza, we used PR8-OVA, an attenuated influenza virus, since even exceedingly low doses of PR8 can be lethal to DBA/1J mice (Figure 4). Consistent with these findings, mice of a related strain, DBA/2J, are highly susceptible to influenza [39–42], and DBA/1J mice are more susceptible than BALB/c mice to a H3N2 influenza virus strain [43]. Notably, PR8 infection is often studied in C57BL/6 and BALB/c mice [44]. The cause of increased susceptibility of DBA mice to influenza infection is not known, but is likely related to multiple genetic factors known to be important in an anti-influenza immune response, including MHC haplotypes [45, 46]. Using PR8-OVA in DBA/1J mice may enable future studies to solve this mystery.

In conclusion, we demonstrate a novel modest requirement for PAD2 in the abnormal anti-collagen autoantibody response in CIA and in the normal antiviral antibody response to influenza. Future investigation is needed to define the mechanisms behind this requirement and to utilize these findings to improve human health.

## Data Availability

Data will be provided upon request to the corresponding author.
